# Twenty-Third Annual Session of the Georgia State Dental Society

**Published:** 1891-06

**Authors:** 


AMERICAN JOURNAL

—OF—

DENTAL SCIENCE.

VOL. XXV. BALTIMORE, JUNE, 1891. NO. 2.

ARTICLE I.

### TWENTY THIRD ANNUAL SESSION OF THE GEORGIA STATE DENTAL SOCIETY.

WAS HELD IN THE ASSEMBLY ROOM AT ST. SIMON'S HOTEL, ST. SIMON'S ISLAND, GEORGIA, ON THE 19TH OF MAY, 1891.

### MORNING SESSION.

The meeting was called to order ten a. m., with President R. B. Adair in the chair.

The exercises were opened with prayer by the Rev. E. Z. F. Golden of Brunswick.

After which the President introduced Hon. W. E. Kay, of Brunswick, who delivered the welcome address, as follows:

*Mr. President, Members of the State Dental Society, Ladies and Gentlemen*:—It has been my pleasure on several occasions to extend on the part of the City of Brunswick, the welcome our people so heartily feel to visiting bodies, but never before, in extending such welcome, have I felt the element of fear so mixed up with respect as towards the body I now behold.

I am here to bid you welcome to St. Simon's, may the breezes of the ocean and the rolling of the surf refresh your bodies that your minds may be spurred on to a higher degree of intellectual thought and deliberation. Your profession, though almost new, is yet rapidly taking front rank about the learned scientific bodies and combines the elements of philanthrophy with health.

On the part of the people of Brunswick I bid you welcome; a welcome that comes from the heart and is seconded by the head. May your stay be so beneficial that as each year recurs it will be a pleasure to extend to you as individuals, if not as a body, a joyful return to these life-giving, hospitable shores. [Applause.]

The President—The address of welcome by Mr. Kay will be responded to by Prof. Coyle, as follows:

*Mr. President, Ladies and Gentlemen:*—Four years ago as we passed through your city on our way to Cumberland Island, our Society was received with most bountiful hospitality. This made a deep impression on the members of our society because it was the first time that a municipality had in a public way, officially extended a welcome to this society. It conveyed to us the idea that you at least appreciated the importance of our work, in attempting to advance our profession. Because you were the first to so recognize us, we will ever hold in grateful remembrance, the citizens of Brunswick, and carry away with us pleasant recollections of their hospitality. [Applause.]

The report of the Executive Committee was received and adopted.

The following letter was read by the Secretary:

"DR. R. B. ADAIR, *President*:—Please say to the Georgia Dental Society that we hope to meet all, every one of them, at the Southern next August 11th, at Morehead City.

"The great merits of this place as a summer resort, together with the untiring committee of arrangements, Col. J. W. Selby will tell you about if an opportunity is offered him.

"Come! all come! bring your wives, mothers, mothers-in-law, also sisters, cousins and aunts. We have to decide about our Southern Dental Home and need the aid of the ladies.

Signed—G. S. F. WHITE, President,

Southern Dental Association."

The Secretary also read a letter from the National Association of Dental Examiners, asking delegates from the Georgia Association to its annual meeting at Saratoga Springs, N. Y., on Monday, August 3d, 1891, at ten a. m.

The Secretary was requested to acknowledge receipt of the communications just read.

At the close of the reading, upon motion, the meeting adjourned until eight p. m.

EVENING SESSION.

Meeting was called to order at eight p. m., with President Adair in the chair.

The President.—Dr. Browne will open the discussion of Dr. Johnson's paper.

Dr. Browne.—I dislike to undertake to discuss the paper unless I could find something in it to which I feel opposed. [Dr. Browne here illustrates his remarks by drawings on the black-board showing the method he sometimes used in putting in one bicuspid.] This method is most frequently used for young ladies where the mouth is otherwise perfect. I said this simply to open the discussion, as I want to hear this question talked about. It is an important thing to us and it is the most satisfactory dentistry that I know of.

I have had but little experience with removable bridges in the way they are made by others. My removable bridges, as you know, have been made with rubber. They are strong and useful and not easily broken.

Dr. White.—I had a patient who had an upper and lower bridge made in New Jersey. It was the dirtiest and most filthy mouth-piece I ever saw. I prescribed a disinfectant mouth wash and remedied the case considerably. I thought the work very poorly done. The roots were loose and the gums diseased, and I think the use of a disinfectant absolutely necessary.

Dr. Frank Holland.—I would like to ask Dr. White what he considers the best disinfectant mouth wash.

Dr. White.—I use carbolic acid and think same the safest disinfectant in the following proportions: Four drachms carbolic acid; three ounces of glycerine; three ounces of water, use on tooth brush.

Dr. Holland.—I find glycerine the best for treating mucous tissue. Glycerine and tannic acid makes a fine application for mucous tissue.

Dr. Frank Holland spoke on the Morrison crown and condemned same. He advocated the Logan as the strongest crown. He asked if anyone had used a Parmly-Brown crown.

Dr. Colding.—I have used a few of them in cases where I found the Logan impracticable.

Dr. Bouton.—I would like to inquire how old Dr. Morrison is, as the crown they are calling by his name is at least fifty and, perhaps, a hundred years old.

Dr. Frank Holland explained why he called it the Morrison crown. He stated that Dr. Atkinson said Morrison invented it, but the journals prove it was made in 1835.

Dr. Browne.—I have recently put in one mouth twenty-three (23) gold crowns, all the way round with satisfactory results. The patient was a gentleman with a heavy mustache.

Dr. Tignor.—My objections to bridges is based upon the ground that I fear they will break down and prove hard to keep in a sanitary condition.

Dr. Carpenter.—I endorse Dr. Johnson's paper. I would like to ask Dr. Tignor if he does not think the same people smelt worse than the bridges. I endorse and believe bridges to be one of the finest achievements of dentistry when properly made. There is no chance for infection when properly made.

Dr. Holland.—There is a doctor present who had a bridge inserted by Dr. Crenshaw, of Atlanta, which is giving perfect satisfaction. I have seen other cases, one that has been in five years and which is giving perfect satisfaction, and these only touch the gum. Dr. Simmons states that the bridge was comfortable, and he can bite as well with it as any tooth in his head. He has worn same for about two years.

Subject passed.

### OPERATIVE DENTISTRY.

The President:—Dr. E. F. Adair's paper on operative dentistry will now be read.

Dr. Osborn in opening the discussion said, I agree with the gentleman that the reason so many fail is that they do not know how to put the filling in.

Dr. White.—I think it impossible to make a good filling at the cervical wall with cohesive gold, and always start those cavities with soft gold; I think it is a mistake to use tin.

Dr. Holland.—Dr. White, you say you do not believe that cohesive gold can be malleted to the cervical wall without cracking the margins. I claim that I can put cohesive gold anywhere without cracking the tooth and to prove that, I will fill a tooth with frail enamel margins and not crack them. I claim that it is impossible to condense soft gold in approx cavities.

Dr. White.—I believe strongly in separations.

Dr. Holland.—I do not believe in such separations. I have some in my own mouth and find them very annoying.

Dr. R. B. Adair.—I do not believe in large gold contours or cohesive gold, but prefer to start in deep cavities with copper amalgam, then soft gold, and finish with cohesive gold.

Dr. Carpenter.—I believe cohesive gold superior in some cases to soft, but believe strongly in soft gold in some places. I would ask the dentists from Macon if they do not see fillings of soft gold put in by Dr. Emerson, in Macon, way back in the fifties, and still in good condition.

Dr. White.—I want to put myself right as to cohesive gold. I use it as well as soft gold, and in some cases it is imperative.

On motion meeting adjourned.

---

MAY 20th, 1891.

The convention was called to order at half past two o'clock p. m., by the President.

Reading of minutes of last regular meeting at Gainesville dispensed with, and referred to a committee consisting of Dr. Golding, Drs. Williams and Ryder for examination and approval.

Dr. C. K. Chapman, of Americus Ga., and Dr. W. S. Simmons, of Guyton, Ga., were elected members.

Upon motion, the Treasurer, Dr. Lowrance, was instructed to embody in his report as treasurer such members who had failed to pay their yearly dues, so the Secretary could erase their names from the roll, and be dropped from the privileges of the Society.

Dr. H. S. Colding exhibited and explained several new appliances, which were as follows:

Dr. W. G. Browne's New Dental Engine, Dental Engine Mallet, Disk Carrier, Parchment Disks and Strips, exhibited by Walker G. Browne's Dental Depot. Also new Duplex Cord Dental Engine, and Gasoline Dental Forge.

Exhibited by S. S. White Dental Manufacturing Company: Partz Acid Gravity Battery, new machine made

Stone Burs, Flexo-Separating Files, Evans Root Dryer, Bromville Engine Mallet, improved No. 1 Mellotte Gold Crown Dies, Absorbent Cotton Rolls.

Holiday Brothers Exhibited 2 Justi's Root Trimmers, 1 Justi's Articulator, 1 Davidson Separator, 1 Werwick Matrix, 1 Werwick Improved Cup, 1 Rowe Disk Carrier.

Dr. H. H. Johnson exhibited his new universal Cervical Clamp.

Committee on Publication consisting of Drs. Coyle, Wardlaw and Catching, reported that type had been set up for the ten thousand copies of the address prepared and read at Gainesville by Dr. B. H. Catching, but reported that no printing of the ten thousand copies had been done.

While waiting for the report of the committee, Dr. Adair said: "I hold in my hand a tooth taken from the mouth of a little negro and placed in the mouth of a beautiful young lady in Gainesville by Dr. Chappell, but a few days afterwards it came loose, and she was seen by Dr. Genese, of Baltimore, who replanted it. It came out the third time, and I replanted it and put a splint on it and kept it there three months. I took the splint off, and I suppose it stayed in her mouth about three months after the splint was taken off and then fell out.

Here is a diagram specimen of bridge work done at Gainesville by Dr. Atkinson, which is giving universal satisfaction.

There were quite a number of other skillful operations performed at that time which are proving beneficial.

I hold in my hand a tooth containing a little gold filling which was put in fifty-four years since, said to have been put in by young Dr. Johnson, who practiced in Savannah. This is as perfect a filling and has as perfect a margin as I have ever seen.

Here is something else I would like to call the attention of young men to: Years ago I went to New York and went into the office of Dr. Atkinson, and he was operating on Mrs. Senator Plum, putting in a crown and gold filling, and after finishing it off nicely he took something and cleaned the tooth off. I asked him what it was, and he said it was "elixir vitriol." I took aromatic sulphuric acid and immersed a tooth in it ten days, and it did not effect it one particle. I put a tooth in lactic acid and you can bend that tooth now. I do not think it will do to use lactic acid and acetic acid on teeth at all. You can apply elixir vitrol and sulphuric acid, which I consider very good. Dr. Atkinson said that sulphuric acid and pumice stone is one of the best solutions he ever used for cleaning teeth.

Dr. Browne.—I have used aromatic sulphuric acid and have never seen any damaging effect to the teeth.

Dr. Colding.—I have used the same thing mixed with pumice stone and have seen no ill effect at all from its use, and use it especially for removing green stains from children's teeth.

Dr. McElhaney.—I have used it twenty-five years and have seen no damaging effect, but it is best not have it too strong.

### DISCUSSION OF COPPER AMALGAM.

Dr. White.—I have two boxes in my office which have never been opened. I have never tried it. I think Dr. Roach knows something about it.

Dr. McElhaney.—When I first commenced to practice, 25 years ago, the first amalgam used was Lawrence's said to have copper in it. When copper amalgam first came out, about eight (8) years ago, I met Dr. Spaulding, of Indiana, who had a brother who was practicing in Paris, and he gave me a few samples of amalgam, and I did not know how to make use of same until I got instructions to heat it in a spoon until it became soft. My first experience was in the mouth ofa lady who had very white teeth, and which have been serviceable for about seven years,and I have seen no failure as yet. I began to use Rodgers'

amalgam brought from England. I began to notice a bright shade on same, I noticed it in mouths that had a good deal of acid in them, and it would cup out. I am satisfied it is acid and nothing else. And another thing; if this amalgam will turn a bronze color it will never cup out. For the last two years I have dropped off using it. I think near the margin of the gum and labial surfaces of molar teeth if it will turn a bronze color it will save teeth better than anything else. You do not see any decay under the filling.

Dr. Holland.—Dr. McElhaney, what reason have you to believe it is acids instead of alkalies, that causes the cupping?

Dr. McElhaney.—By noticing softness of teeth in the mouth. Persons who do not like vinegar have acid in their mouth as well as those that do. But when a man goes in for copper amalgam he must study the condition of the teeth. Soft, chalky, brittle, whitish teeth you can save with copper amalgam when you cannot save with anything else in the world.

Dr. White.—I cannot for the life of me see that acid condition of mouths should have such an effect on materials. I think there must be some other agent rather than acid mouths. Some copper amalgam was sent me a few years ago by a brother dentist who said he wanted me to use it, but I did not do so. A patient of his came to me a few years afterwards and I found the second bicuspid so much discolored that discoloration had covered the whole tooth. The amalgam was not discolored but the entire tooth was discolored; and I became very much prejudiced toward copper amalgam.

Dr. Browne.—Do you know, Dr. White, that it was copper amalgam?

Dr. White.—I do not know that it was copper amalgam, but I think it was. This color had permeated even the enamel of the tooth. I dropped off entirely the use of copper amalgam; I do not know whether I am right or not.

Dr. Browne.—I think you are wrong, because I have watched the effect of it for several years, and have yet to see a filling of copper amalgam discolor a tooth. I think it a question that we all had better give attention to, and get further light on, for further light is needed; and if we will go to work and watch the effect of its various symptoms I think we have the promise in it of one of the best filling materials we have.

Dr. Colding,—I have had a little experience from the same dentist that Brother White speaks of. It is not like the white amalgam, it is without doubt the dirtiest mess I ever saw, I would not use it. I asked him how he made it and he said he made it himself with a galvanic battery.

Dr. McElhaney.—I have seen but one gentleman who takes the position of Dr. White, that it is not acid—Dr. Chapein, of Philadelphia—he has made copper amalgam and he has noticed this cupping out; he says that he thinks he can make amalgam that will not cup out. Most gentlemen think that it is the way it is made.

Dr. Colding.—Where you find it cupping out, it is always bright, which I think shows acid action.

Dr. White.—I should think it is very easy for us to satisfy ourselves on that score, and if it is acid we can ascertain that out of the mouth as well as in it. Speaking of it cupping out, the amalgam I have been talking about I think was copper amalgam. I am satisfied that no doctor would use it, for I never had such a job in my life as I did when I undertook to get that amalgam out.

Dr. Browne took the chair and Dr. Adair addressed the Convention.

Dr. Adair.—Three years ago I took no stock in copper amalgam. Some friend sent me a sample, and my office boy had a case where copper amalgam could be used, so I decided I would experiment. I took the boy and filled his teeth with it and it was not four hours before it was jet black. And during the next day I asked him how it felt, and he said it felt all right. That has been about two years ago, and I have never seen as pretty a margin in my life; it is in a perfect state of preservation under the gum and everywhere else. Since that time I have been filling with copper amalgam when white chalky teeth are brought to me. My opinion coincides with Dr. Browne's that it preserves the teeth and does not discolor them. I had some put in my mouth the other day under a little gold, and it is comfortable to-day. It was some time before I learned how to manipulate copper amalgam. My experience is that this wasting away is not knowing how to mix it. The more mercury you can get in amalgam the better it is.

Dr. Colding.—I have seen work from the hands of a very eminent practitioner who uses an excess of mercury, and a great deal of the work is failing.

Dr. McElhaney.—A very large contour filling I put in ten years ago had decayed under the filling but was perfectly sound, and I did not want to interrupt the filling way under the gum margin. I filled it with copper filling and it turned green, as it came in contact with gold. The color came up some distance in the tooth.

Dr. Browne.—I am satisfied from the different methods the gentlemen mentioned in manipulating it that the mixing of same has a great deal to do with its success. I am using Buck's amalgam now, and the way I mix it is this: I put it into a bowl and grind it up and add a little mercury.

Dr. Carpenter.—Might it not have affected it somewhat by grinding it in an iron mortar.

Dr. Browne.—Yes, it might have affected it a little.

Dr. Tignor.—I have used copper amalgam somewhat, and find it is a very good thing to preserve teeth, and to be used on side of teeth up near the gum.

On motion the subject was passed.

### PATHOLOGY AND MICROSCOPY.

No paper; subject passed.

*To be continued.*

